# Triglyceride to high-density lipoprotein cholesterol (HDL-C) ratio and arterial stiffness in Japanese population: a secondary analysis based on a cross-sectional study

**DOI:** 10.1186/s12944-018-0776-7

**Published:** 2018-05-29

**Authors:** Chi Chen, Jia-Lin Dai

**Affiliations:** 1 Department of Immunology and Microbiology, Guiyang College of Traditional Chinese Medicine, 84# ShiDong Road, Guiyang, 550001 Guizhou China; 2 School of forensic medicine, Guizhou Medical University, 2# Beijing Road, Guiyang, 550001 Guizhou China

**Keywords:** Triglyceride, High-density lipoprotein cholesterol, Brachial-ankle pulse wave velocity, Association

## Abstract

**Background:**

Previous studies have revealed that triglyceride to high-density lipoprotein cholesterol (HDL-C) ratio (henceforth TG/HDL-C) is one of major risk factors of cardiovascular diseases, insulin resistance and metabolism syndrome. However, there are fewer scientific dissertations about the correlation between TG/HDL-C and bapWV. This study was undertaken to investigate the relationship between Triglyceride (TG) to high-density lipoprotein cholesterol (HDL-C) ratio and brachial-ankle pulse wave velocity (baPWV) in Japanese.

**Methods:**

The present study was a cross-sectional study. 912 Japanese men and women, aging 24−84 years old, received a health medical a health check-up program including the results from baPWV inspection and various standardized questionnaire in a health examination Center in Japan. Main outcome measures included TG/HDL-C ratio, baPWV, fatty liver, postmenopausal status. Abdominal ultrasonography was used to diagnose fatty liver. Postmenopausal state was defined as beginning 1 year after the cessation of menses. It was noted that the entire study was completed by Fukuda et al., and uploaded the data to the DATADRYAD website. The author only used this data for secondary analysis.

**Results:**

After adjusting potential confounders (age, sex, BMI, SBP, DBP, AST, ALT, GGT, uric acid, fasting glucose, TC, LDL, eGFR, smoking and exercise status, fatty liver, alcohol consumption and ABI), non-linear relationship was detected between TG/HDL-C and baPWV, whose point was 5.6. The effect sizes and the confidence intervals on the left and right sides of inflection point were 12.7 (1.9 to 23.5) and − 16.7 (− 36.8 to 3.3), respectively. Subgroup analysis showed, in participants with excessive alcohol consumption (more than 280 g/week), that TG/HDL-C had a negative correlation with BAPWV (β = − 30.7, 95%CI (− 53.1, − 8.4)), and the P for interaction was less than 0.05,

**Conclusion:**

The relationship between TG/HDL-C and baPWV is non-linear. TG/HDL-C was positively related with baPWV when TG/HDL-C is less than 5.6. In addition, while the trend is opposite in excessive alcoholic subjects.

## Background

Brachial-ankle pulse wave velocity (baPWV) is served as an indicator to quantify arterial stiffness [1]. As an independent risk factor of cardiovascular events, baPWV is used in clinical for early evaluating the functions and structural changes of vascular wall [2, 3]. Despite of the fact that western countries have not fully accepted baPWV, more and more publications on this research methodology came from these countries since 2009 [1]. Atherosclerosis Risk in Communities (ARIC) study and the Bogalusa Heart Study, the two large-scale studies in U. S, have used baPWV as indicator to assess arterial stiffness [4, 5].

Previous studies have revealed that triglyceride to high-density lipoprotein cholesterol (HDL-C) ratio (henceforth TG/HDL-C) is one of major risk factors of cardiovascular diseases, insulin resistance and metabolism syndrome [6–11]. Some scholars consider that TG/HDL-C can better predict vascular risk than either does [12]. However, there are fewer scientific dissertations about the correlation between TG/HDL-C and bapWV. In only a few dissertations [13–15], as authors used the TG/HDL for analyzing categorical variables. In addition, they used GLM as a sole method of data analysis, in which the independent variables and dependent variables must be linear. But in biomedical research, connection between exposures and outcomes may be non-linear. In that case, researchers need a more effective method to deal with non-linear relationship.

In this study, we performed a secondary data analysis based on an existing data that comes from the published paper [16]. In original paper, the author have investigated the correlation between γ-glutamyltranspeptidase and baPWV. While in secondary analysis, TG/HDL was used as independent variable, and outcome variable and other covariates are consistent with those in the original.

## Methods

### Data source

We obtained data from ‘DATADRYAD’ database (www.Datadryad.org). This website permitted users to freely download the raw data. According to Dryad Terms of Service, we cited Dryad data package in the present study. (Dryad data package: Fukuda T, Hamaguchi M, Kojima T, Ohshima Y, Ohbora A, Kato T, Nakamura N, Fukui M (2014) Data from: Association between serum γ-glutamyltranspeptidase and atherosclerosis: a population-based cross-sectional study. Dryad Digital Repository. 10.5061/dryad.m484p). Variables included in the database file were as follows: age, body mass index (BMI), diastolic blood pressure (DBP), systolic blood pressure (SBP), alanine aminotransferase (ALT), aspartate transaminase (AST), γ-glutamyltranspeptidase (GGT), uric acid, fasting glucose, total cholesterol (TC), low density lipoprotein (LDL), estimated glomerular filtration rate (eGFR), baPWV, sex, smoking status, exercise, fatty liver disease, menopausal status, alcohol consumption, ankle-brachial index (ABI), high-density lipoprotein cholesterol (HDL-C) and triglyceride (TG),

### Study population

Takuya Fukuda et al. [16] completed the entire study. In order to allow to understand the entire research process more clearly, we have outlined the steps of the study here. The specific details are described in the original. Reported by Takuya Fukuda et al. They conducted a cross-sectional study at Medical Health Checkup Center of Murakami Memorial Hospital, Gifu city, Japan from March 2004 to December 2012. Participants received a medical health check-up programme including pulse wave velocity and abdominal ultrasonography. A total of 1, 445 participants were recruited and selected according to exclusion standard. Exclusion standards: (1) participants received hormone replacement therapy, (2) participants took oral contraceptives, (3) Hepatitis B virus antigen and hepatitis C virus antigen was positive; (4) The participants were in gestational age, (5) ankle-brachial index (ABI) was less than 0.95. Researchers obtained information (values) of baPWV, TG/HDL and other covariants at baseline. This study was performed by Japanese researcher Takuya Fukuda and Masahide Hamaguchi, et al. at the Medical Health Checkup Center of Murakami Memorial Hospital, Gifu city, Japan. In the previously published article [16], Takuya Fukuda, et al. has clearly stated that: the study was conducted in accordance with the Declaration of Helsinki. Informed consent was obtained from all Participants.

#### Measurement of baPWV, TG/HDL-C and other covariants

baPWV and ABI were measured using an automatic waveform analyzer (Colin Medical Technology, Komaki, Japan). The subjects took the supine position and rested in a quiet and suitable temperature room for 5 min, and then placed ECG electrodes and heart sound microphone on both writs and the left edge of the sternal border respectively. Cuffs connected with a plethysmographic sensor and an oscillometric pressure sensor were wrapped on the brachia and ankles. Since then, Takuya Fukuda et al. calculated the path lengths from the suprasternal notch to the brachium (Lb) and from the suprasternal notch to the ankle (La), and then automatically obtained the delay time from the ascending point of the brachial waveform to the ascending point of each ankle waveform (DTba). Finally, they calculate baPWV by formula (La-Lb) / DTba. The measurement and assessment of TG/HDL and other covariants were described in detail in the original.

### Statistical analysis

Continuous variables were expressed as mean ± standard deviation (normal distribution) or median (quartile) (skewed distribution), and categorical variables were expressed in frequency or as a percentage. The One-Way Anova (normal istribution), Kruscal Whallis H (skewed distribution) test and chi-square tests (categorical variables) were used to determine any statistical differences between the means and proportions of the groups. Univariate linear regression model was used to evaluate the associations between TG/HDL and baPWV. Both non-adjusted and multivariate adjusted models were listed in the paper. According to the recommendation of STROBE statement, we simultaneously showed the results of unadjusted, minimally adjusted analyses and those from fully adjusted analyses. Whether the covariances were adjusted determined by the following principle: when added to this model, changed the matched odds ratio by at least 10% [17]. Besides, we also used generalized additive model (GAM) to identify the non-linear relationship. If the non-linear correlation was observed, a two-piecewise linear regression model was performed to calculate the threshold effect of the TG/HDL on baPWV in terms of the smoothing plot. When the ratio between baPWV and TG/HDL appears obvious in smoothed curve, recursive method calculates automatically the inflection point, where the maximum model likelihood will be used [18]. The subgroup analyses were performed using stratified linear regression models. The modification and interaction of subgroup were inspected by the likelihood ration test. All of the analyses were performed with the statistical software packages R (http://www.R-project.org, The R Foundation) and EmpowerStats (http://www.empowerstats.com, X&Y Solutions, Inc., Boston, MA). *P* values less than 0.05 (two-sided) were considered statistically significant.

## Results

### The selection of participants

Of the 1445 participants, 533 participants were excluded from this study. In 533 excluded subjects, 433 received medcation, 1 took oral contraceptive, 66 received hormone replacemet therapy, 26 with hepatitis B and hepatitis C antigen was positive, 1 was in gestational age, and 6 had ABI less than 0.96, leaving 912 subjects for data analysis.

### Baseline characteristics of participants

The average age of the participants was 51.1 ± 9.6 years old,and about 64.9% of them are male. Baseline characteristics were listed in Table 1. There was no statistically significant difference in age among different TG/HDL-C groups. Compared with high level (Q4) of TG/HDL-C group, patients had a significantly lower BMI,SBP,DBP, AST, ALT, GGT, uric acid, fasting glucose, TC, LDL, eGFR and baPWV, ABI in other three groups (Q1-Q3).

**Table 1:** Baseline Characteristics of participants

TG/HDL-C	Q1	Q2	Q3	Q4	*P*-value	*P*-value*
Number	228	228	228	228		
AGE (years, mean ± sd)	50.8 ± 10.0	51.8 ± 9.7	51.2 ± 9.0	50.7 ± 9.6	0.638	0.600
BMI (kg/m2, mean ± sd)	21.5 ± 2.4	22.6 ± 2.8	23.6 ± 3.4	24.7 ± 2.9	< 0.001	< 0.001
SBP (mmHg, mean ± sd)	114.1 ± 13.4	118.5 ± 15.0	123.4 ± 15.1	124.9 ± 14.0	< 0.001	< 0.001
DBP (mmHg, mean ± sd)	71.4 ± 9.1	74.9 ± 9.8	78.3 ± 10.2	80.0 ± 8.7	< 0.001	< 0.001
AST(IU/L, mean ± sd)	19.3 ± 6.2	19.8 ± 6.0	20.9 ± 9.4	23.4 ± 9.5	< 0.001	< 0.001
ALT(IU/L, mean ± sd)	16.0 (12.0–20.0)	17.0 (13.0–23.0)	20.0 (15.0–28.0)	24.0 (18.0–35.0)	< 0.001	< 0.001
GGT (IU/L, mean ± sd)	14.0(11.0–17.0)	18.0(14.0–25.2)	20.0(15.0–33.0)	24.0 (19.0–41.0)	< 0.001	< 0.001
Uric acid (mg/dl, mean ± sd)	4.5 ± 1.2	4.9 ± 1.3	5.5 ± 1.2	6.1 ± 1.2	< 0.001	< 0.001
Fasting glucose (mg/dL, mean ± sd)	93.1 ± 8.3	96.2 ± 10.7	100.8 ± 20.1	102.1 ± 12.4	< 0.001	< 0.001
TC (mg/dL, mean ± sd)	202.7 ± 34.6	205.8 ± 34.4	210.3 ± 34.7	220.6 ± 37.7	< 0.001	< 0.001
LDL (mg/dL, mean ± sd)	115.2 ± 29.2	126.7 ± 29.0	133.7 ± 29.7	136.6 ± 34.4	< 0.001	< 0.001
eGFR(mL/min/1.73 m^2^, mean ± sd)	73.9 ± 13.2	70.8 ± 12.2	69.1 ± 10.7	67.8 ± 11.1	< 0.001	< 0.001
baPWV (cm/s, mean ± sd)	1344.9 ± 220.6	1410.0 ± 231.8	1435.0 ± 232.1	1473.2 ± 279.9	< 0.001	< 0.001
SEX (n,%)					< 0.001	–
Male	76 (33.3%)	141 (61.8%)	173 (75.9%)	202 (88.6%)		
Female	152 (66.7%)	87 (38.2%)	55 (24.1%)	26 (11.4%)		
Current smokig (n,%)					< 0.001	–
None	209 (91.7%)	181 (79.4%)	166 (72.8%)	159 (69.7%)		
Current	19 (8.3%)	47 (20.6%)	62 (27.2%)	69 (30.3%)		
Ex-Smoking (n,%)					< 0.001	–
No	162 (71.1%)	126 (55.3%)	91 (39.9%)	82 (36.0%)		
Yes	66 (28.9%)	102 (44.7%)	137 (60.1%)	146 (64.0%)		
Regular Exercise (> 1 week) (n,%)					0.003	–
No	165 (74.3%)	172 (76.8%)	187 (82.7%)	195 (87.1%)		
Yes	57 (25.7%)	52 (23.2%)	39 (17.3%)	29 (12.9%)		
Fatty liver (n,%)					< 0.001	–
None	210 (92.1%)	193 (84.6%)	141 (62.1%)	102 (44.7%)		
Yes	18 (7.9%)	35 (15.4%)	86 (37.9%)	126 (55.3%)		
Post-Menopausal (n,%)					0.118	–
No	75 (49.3%)	33 (37.9%)	18 (32.7%)	12 (46.2%)		
Yes	77 (50.7%)	54 (62.1%)	37 (67.3%)	14 (53.8%)		
Alcohol consumption (n,%)					< 0.001	–
< 40 (g/week)	174 (76.3%)	140 (62.2%)	131 (58.2%)	136 (61.5%)		
≤40–140	27 (11.8%)	48 (21.3%)	36 (16.0%)	39 (17.6%)		
> 140–280	18 (7.9%)	21 (9.3%)	27 (12.0%)	22 (10.0%)		
> 280	9 (3.9%)	16 (7.1%)	31 (13.8%)	24 (10.9%)		
ABI	1.3 ± 0.8	1.2 ± 0.1	1.2 ± 0.1	1.2 ± 0.1	< 0.001	

*ALT* alanine aminotransferase, *AST* aspartate transaminase, *baPWV* brachial-ankle pulse wave velocity, *BMI* body mass index, *eGFR* estimated glomerular filtration rate, *GGT* γ-glutamyltranspeptidase, *HDL-C* high-density lipoprotein cholesterol, *LDL-C* low-density lipoprotein cholesterol, *SBP* systolic pressure, *DBP* Diastole pressure, *Tg* triglyceride, *TC* total cholesterol, *ABI* ankle-brachial index

### Univariate analysis

The results of univariate analysis were shown in Table 2. The results of univariate analysis showed that age, BMI, SBP, DBP, AST, ALT, fasting glucose, GGT, uric acid, TC, alchohol consumption (> 280 g/weed), LDL-C, TG/HDL-C and fatty liver were correlated with higher baPWV. We also found that BMI, smoking, ABI and exercise status were not associated with baPWV, whereas eGFR was negatively associated with higher bapWV.

**Table 2:** The results of univariate analysis

	Statistics	Effect size (β)	*P* value
Sex			
Male	592 (64.9%)	ref	
Female	320 (35.1%)	−49.4 (−82.8, −16.1)	0.004
Age	51.1 ± 9.6	12.9 (11.5, 14.4)	< 0.001
BMI	23.1 ± 3.1	5.0 (−0.2, 10.1)	0.058
SBP	120.2 ± 15.0	8.4 (7.5, 9.4)	< 0.001
DBP	76.1 ± 10.0	11.3 (9.9, 12.7)	< 0.001
AST	20.9 ± 8.1	3.4 (1.4, 5.4)	0.001
Fasting glucose	98.1 ± 14.1	4.2 (3.1, 5.3)	< 0.001
ALT	22.7 ± 14.3	1.5 (0.4, 2.6)	0.009
GGT	25.4 ± 24.4	1.0 (0.4, 1.7)	0.002
Uric acid	5.3 ± 1.4	22.8 (11.3, 34.3)	< 0.001
TC	209.8 ± 36.0	0.7 (0.3, 1.2)	0.002
Alcohol consumption			
≤40 (g/week)	581 (64.6%)	ref	
40–140	150 (16.7%)	−7.1 (−51.3, 37.1)	0.753
140–280	88 (9.8%)	13.0 (−42.2, 68.1)	0.645
> 280	80 (8.9%)	50.8 (−6.7, 108.3)	0.084
LDL-C	128.1 ± 31.7	0.7 (0.2, 1.2)	0.010
TG/HDL-C	2.2 ± 2.1	14.9 (7.2, 22.5)	< 0.001
Curren Smoking			
None	715 (78.4%)	ref	
Current	197 (21.6%)	−0.2 (−39.0, 38.7)	0.994
Ex-Smoking			
None	461 (50.5%)	ref	
Yes	451 (49.5%)	25.4 (−6.5, 57.4)	0.119
Regular Exercise (> 1 week)			
No	719 (80.2%)	ref	
Yes	177 (19.8%)	16.7 (−23.2, 56.5)	0.413
Fatty liver			
No	646 (70.9%)	ref	
Yes	265 (29.1%)	93.7 (59.0, 128.5)	< 0.001
eGFR	70.4 ± 12.0	−6.4 (−7.6, − 5.1)	< 0.001
ABI	1.2 ± 0.4	30.7 (−8.9, 70.3)	0.129

### The results of relationship between TG/HDL-C and baPWV

We used univariate linear regression model to evaluate the associations between TG/HDL and baPWV. Meanwhile, we showed the non-adjusted and adjusted modles in Table 3. In crude model, TG/HDL showed positive correlation with baPWV (β = 14.9, 95% confidence interval (CI): 7.2 to 22.5, *P* < 0.001). In minimally adjusted model (adjusted age, sex), the result did not have obvious changes (β = 12.7, 95%CI: 5.8 to 19.6, *P* < 0.001). However, we did not detect the connection in fully adjusted model (β = 1.8, 95%CI: -5.0 to 8.5, *P* = 0.609). For the purpose of sensitivity analysis, we also handled TG/HDL-C as Categorical variable (Quartile), and found that the same trend was observed as well(p for trend was 0.171).

**Table 3:** Relationship between TG/HDL-C and baPWV in different models

Variable	Crude model (β, 95%CI, P)	Minimally adjusted model(β, 95%CI, P)	Fully adjusted model(β, 95%CI, P)
TG/HDL-C	14.9 (7.2, 22.5) < 0.001	12.7 (5.8, 19.6) < 0.001	1.8 (− 5.0, 8.5) 0.609
TG/HDL-C(quartile)			
Q1	Ref	Ref	Ref
Q2	65.1 (20.6, 109.6) 0.004	41.4 (2.2, 80.5) 0.039	28.7 (−5.5, 63.0) 0.101
Q3	90.1 (45.6, 134.6) < 0.001	69.2 (28.9, 109.6) 0.001	14.2 (−23.3, 51.7) 0.459
Q4	128.3 (83.9, 172.8) < 0.001	108.1 (66.3, 149.9) < 0.001	35.1 (−5.2, 75.4) 0.088
P for trend	< 0.001	< 0.001	0.171

Crude model: we did not adjust other covariants

Minimally adjusted model: we adjusted age and sex

Fully adjusted model: we adjusted age, sex, BMI, SBP, DBP, AST, ALT, GGT, uric acid, fasting glucose, TC, LDL, eGFR, smoking and exercise status, fatty liver, alcohol consumption, ABI

*CI* confidence interval, *Ref* reference

### The analyses of non-linear relationship

**Fig. 1:**
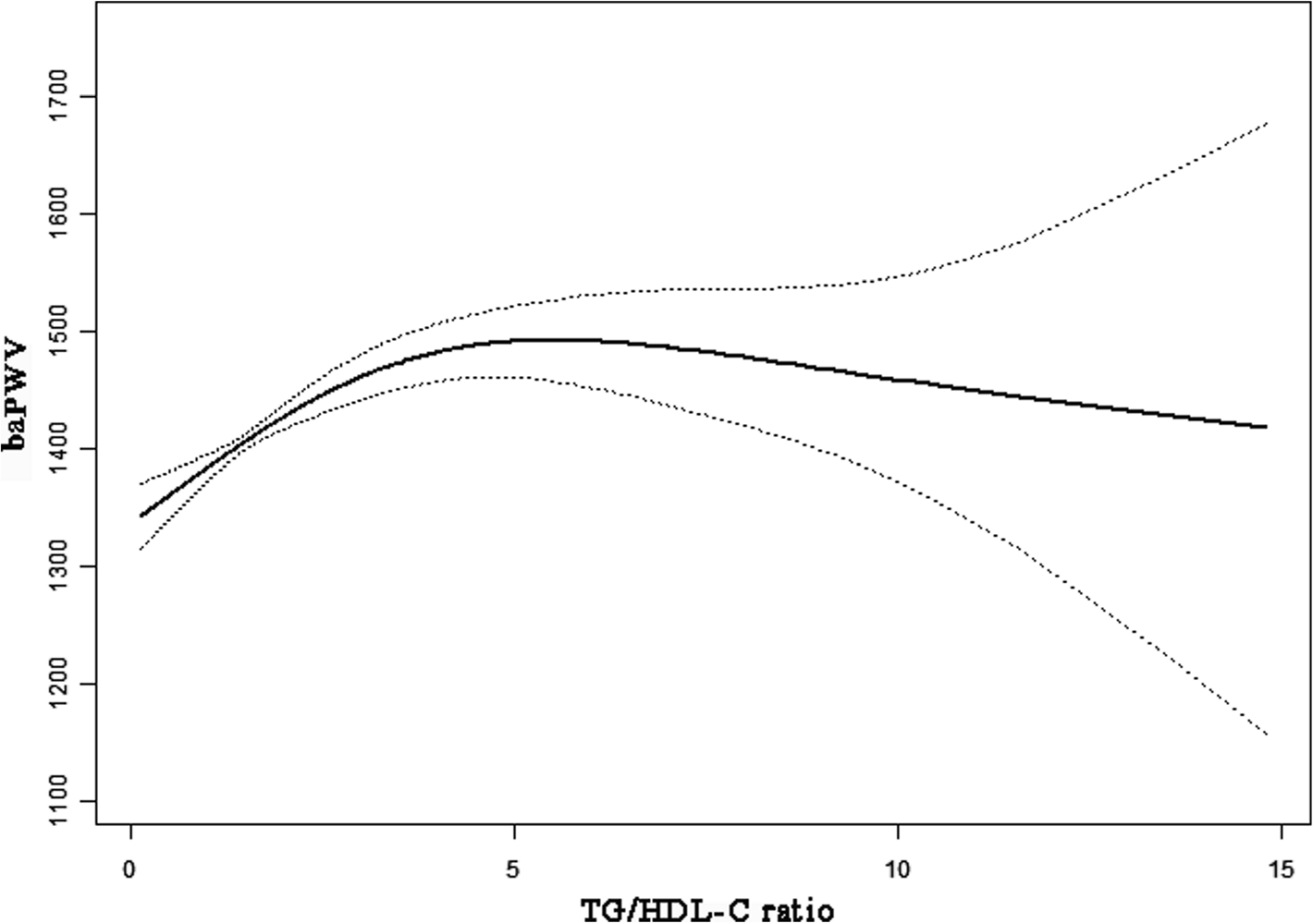
The relationship between TG/HDL-C and baPWV. A nonlinear relationship between them was detected after adjusting for age, sex, BMI, SBP, DBP, AST, ALT, GGT, uric acid, fasting glucose, TC, LDL, eGFR, smoking and exercise status, fatty liver, alcohol consumption and ABI

Because TG/HDL-C was continuous variable, the analyses of non-linear relationship are necessary. In the present study (Fig. 1), we found that the relationship between TG/HDL-C and baPWV was non-linear (after adjusting age, sex, BMI, SBP, DBP, AST, ALT, GGT, uric acid, fasting glucose, TC, LDL, eGFR, smoking and exercise status, fatty liver, alcohol consumption and ABI). By two-piecewise linear regression model, we calculated the inflection point was 5.6. On the left of inflection point, the effect size, 95%CI and *P* value were 12.7, 1.9 to 23.5 and 0.021, respectively. However, we observed no relationship between TG/HDL-C and baPWV on the right of inflection point (− 16.7, − 36.8 to 3.3, 0.102) (Table 4).

**Table 4:** The results of two-piecewise linear regression model

Inflection point of TG/HDL-C	Effect size (β)	95%CI	*P* value
< 5.6	12.7	1.9 to 23.5	0.021
≥5.6	−16.7	−36.8 to 3.3	0.102

Effect: baPWV Cause: TG/HDL-C

Adjusted: age, sex, BMI, SBP, DBP, AST, ALT, GGT, uric acid, fasting glucose, TC, LDL, eGFR, smoking and exercise status, fatty liver, alcohol consumption, ABI

### The results of subgroup analyses

As is shown in Table 5, the test for interactions were significant for alcoholic consumption. (P for interaction = 0.01), while the test for interactions were not statistically significant for age, sex, current smoking, ex-smoking, exercise status, fatty liver, BMI, hypertension and uric acid (P for interaction = 0.85, 0.69, 0.15, 0.31, 0.67, 0.99, 0.50, 0.72 and 0.07, respectively). We observed that there was evidence for an interaction TG/HDL-C and alcoholic consumption. The effect sizes of TG/HDL-C on baPWV showed significant differences in different alcoholic consumption. TG/HDL-C was negatively associated with baPWV in excessive alcoholics (β = − 30.7, 95%CI (− 53.1, − 8.4)). It was noted that Takuya Fukuda et al. collected the menopausal status in raw data, therefore, we also adjusted it in female. Compared with no-adjusted menopausal status (7.2 (− 11.4, 25.8)), however, the effect size of TG/HDL-C on baPWV (8.0 (− 10.5, 26.5)) was not altered after adjusting for menopausal status.

**Table 5:** Effect size of TG/HDL-C on baPWV in prespecified and exploratory subgroups

Characteristic	No of participants	Effect size(95%CI)	P for interaction
Age (year)			0.85
≤60	139	6.0 (− 12.3, 24.3)	
> 60	773	4.1 (− 3.8, 12.0)	
Sex			0.69
Male	592	3.2 (−4.8, 11.2)	
Female	320	7.2 (−11.4, 25.8) 8.0 (− 10.5, 26.5)^a^	
Current smoking			0.15
No	715	7.4 (−1.2, 16.0)	
Yes	197	−4.0 (−17.1, 9.1)	
Ex-Smoking			
No	462	8.6 (−4.0, 21.2)	0.31
Yes	450	0.8 (−8.1, 9.8)	
Regular Exercise (> 1 week)			0.67
No	719	4.6 (−3.3, 12.5)	
Yes	177	0.08 (−20.1, 20.2)	
Fatty liver			0.99
No	646	7.3 (− 2.4, 17.0)	
Yes	265	7.4 (−4.3, 19.1)	
Alcohol consumption			0.01
≤40 (g/week)	581	7.1 (−2.9, 17.0)	
40–140	150	7.0 (−11.3, 25.3)	
140–280	88	−5.2 (−25.4, 15.0)	
> 280	80	−30.7 (−53.1, −8.4)	
BMI			0.50
< 18.5	36	51.9 (−33.8, 137.6)	
> = 18.5, < 23	435	5.2 (−6.5, 16.9)	
> = 23	441	2.8 (−6.5, 12.2)	
Hypertension			0.72
No	790	4.4 (−3.6, 12.3)	
Yes	122	8.4 (−12.3, 29.0)	
Uric acid (tertile)			0.07
Low	298	−6.0 (−25.4, 13.3)	
Middle	308	14.7 (1.3, 28.1)	
High	306	−2.8 (−13.0, 7.3)	

Note 1:Above model adjusted for age, sex, BMI, SBP, DBP, AST, ALT, GGT, uric acid, fasting glucose, TC, LDL, eGFR, smoking and exercise status, fatty liver, alcohol consumption and ABI

Note 2:In each case, the model is not adjusted for the stratification variable

Note 3: ^a^ adjusted menopausal status + age, sex, BMI, SBP, DBP, AST, ALT, GGT, uric acid, fasting glucose, TC, LDL, eGFR, smoking and exercise status, fatty liver, alcohol consumption

## Discussion

The present study was to examine the relationship between TG/HDL-C on baPWV among participants. As is shown in fully adjusted model, TG/HDL-C was not associated with baPWV even analyzed by sensitivity analysis. However, we also found the non-linear relationship between TG/HDL-C and baPWV. The different correlations of TG/HDL-C on baPWV were found on the left and right sides of inflection point (TG/HDL-C = 5.6). TG/HDL-C, as assessed in baseline, was positively associated with baPWV on the left side of inflection point, but the association on the right of inflection was not statistically significant. Interestingly, we also found they have negative correlation with baPWV in participants with excessive drinking.

We conducted a PubMed search simultaneously using the key words‘brachial-ankle pulse wave velocity’and ‘Triglyceride to HDL-C ratio’. Nine scientific papers were retrieved on database as of the end of October 2017, but only three of them were related to our study. In the present study, the result we found using two-piecewise linear regression model is similar to that obtained by Wen JH et al. [19] in a cross-sectional study of apparently healthy individuals. In that study, they used multivariable logistic regression models to calculate the OR of TG/HDL-C on baPWV in 926 men and 572 women. After adjusting potential confounders (age, BMI, SBP, DBP, LDL, fasting glucose, uric acid and eGFR), the OR gradually increased in Q1 to Q4 of TG/HDL-C quartile, and the P for trend was less than 0.05 both in male and female. This suggests that TG/HDL-C is related to baPWV in apparently healthy individuals, however, the adjustment of potential confounders was not sufficient. Such as alcoholic consumption, smoking, exercise status and menopausal status (in female) were not adjusted. Therefore, their conclusions was limited because these confounders mentioned above were closely related with arterial stiffness [20–24]. The other two literatures also reported the positive association of TG/HDL-C with baPWV, although the two studies differ in the study population and research design, the same drawbacks also were found in them [25, 26].

The exploration of subgroup analyses is extremely important for a scientific study [27]. Unfortunately, the above three papers conducted a subgroup analysis only using sex as stratification factor, and they did not test the interaction, which inhibits our exploration of true relationship between TG/HDL-C and baPWV. In the present study, we used age, sex, current smoking, ex-smoking, exercise status, fatty liver, BMI, hypertension, uric acid and alcoholic consumption as stratification variables in which just alcoholic consumption was found. Wakabayashi I [28] reported that alcohol drinking was inversely associated with TG/ HDL-C ratio in middle-aged Japanese men, but in subjects with hypertion and with diabetes, the same trend also was found in Japanese [29–31]. Besides, previous studies also reported that higher alcoholic consumption had significantly higher baPWV compared with those of light to middle alcoholic consumption [32–34]. These findings can be used for explaining the negative association between TG/HDL-C and baPWV observed in excessive alcoholics.

Our study has a number of strengths. First, we not only use the generalized linear model to evaluate the linear relationship between TG/HDL-C and baPWV, but also use the generalized additive model to clarify the nonlinear relationship. GAM has obvious advantages in dealing with non-linear relations and it can handle the non-parametric smoothing and will fit a regression spline to the data. The use of GAM will help us to better discover the real relationships between exposure and outcome. Second, this study is an observational study including unavoidable potential confounding, so we used strict statistical adjustment to minimize residual confounding. Although the previous study reported a linear association between TG/HDL-C and baPWV, we did not detect this relationship in our study after adjusting smoking, alcoholic consumption and other confounding factors which were not adjusted by previous study. Third, the effect modifier factor analysis makes the use of data better. The negative association of TG/HDL-C on baPWV in subjects with excessive alcoholic consumption is found in subgroup analysis.

There are some limitations in our study. First, this study is a analytical cross-sectional study and thus provide only weak evidence between exposure and outcome, and it is difficult to distinguish the cause and effect. Second, as the study population contains only Japanese, it may be not generalisable to other biographic ethic groups. Third, due to raw data limitations, we cannot observe the correlation between insulin resistance and (HDL-C) ratio and arterial stiffness. Similarly, we also cannot investigate the plasma levels of inflammatory markers such as tumor necrosis factor, interleukin, and high-sensitivity C-reactive protein and their possible correlation with (HDL-C) ratio and arterial stiffness.

## Conclusion

The relationship between TG/HDL-C and baPWV is non-linear. TG/HDL-C is positively related with baPWV when TG/HDL-C was less than 5.6. In addition, the negative association between them is found in excessive alcoholic subjects.

## Abbreviations

ABI: Ankle-brachial index
baPWV: Brachial-ankle pulse wave velocity
eGFR: Estimated glomerular filtration rate
LDL: Low density lipoprotein
TC: Total cholesterol
TG/HDL-C: Triglyceride to high-density lipoprotein cholesterol
